# Genomic Insights Into Inbreeding and Adaptive Divergence of Trout Populations to Inform Genetic Rescue

**DOI:** 10.1111/eva.70090

**Published:** 2025-03-20

**Authors:** Donovan A. Bell, Kellie J. Carim, Ryan Kovach, Lisa A. Eby, Craig Barfoot, Sally Painter, Angela Lodmell, Stephen J. Amish, Seth Smith, Leo Rosenthal, Beau Larkin, Philip Ramsey, Andrew R. Whiteley

**Affiliations:** ^1^ Wildlife Biology Program University of Montana Missoula Montana USA; ^2^ Montana Fish Wildlife and Parks Missoula Montana USA; ^3^ U.S.D.A. Forest Service, Rocky Mountain Research Station Aldo Leopold Wilderness Research Institute Missoula Montana USA; ^4^ Confederated Salish and Kootenai Tribes Pablo Montana USA; ^5^ University of Montana Conservation Genomics lab Missoula Montana USA; ^6^ Washington Department of Fish and Wildlife Seattle Washington USA; ^7^ MPG Ranch Florence Montana USA

**Keywords:** genetic rescue, inbreeding, isolation, *Oncorhynchus lewisi*, westslope cutthroat trout

## Abstract

Genetic rescue, specifically translocation to facilitate gene flow among populations and reduce the effects of inbreeding, is an increasingly used approach in conservation. However, this approach comes with trade‐offs, wherein gene flow may reduce fitness when populations have adaptive differentiation (i.e., outbreeding depression). A better understanding of the interaction between isolation, inbreeding, and adaptive divergence in key traits, such as life history traits, will help to inform genetic rescue efforts. Stream‐dwelling salmonids, such as the westslope cutthroat trout (*Oncorhynchus lewisi*; WCT), are well‐suited for examining these trade‐offs because they are increasingly isolated by habitat degradation, exhibit substantial variation in life history traits among populations, and include many species of conservation concern. However, few genomic studies have examined the potential trade‐offs in inbreeding versus outbreeding depression in salmonids. We used > 150,000 SNPs to examine genomic variation and inbreeding coefficients in 565 individuals across 25 WCT populations that differed in their isolation status and demographic histories. Analyses of runs of homozygosity revealed that several isolated WCT populations had “flatlined” having extremely low genetic variation and high inbreeding coefficients. Additionally, we conducted genome scans to identify potential outlier loci that could explain life history differences among 10 isolated populations. Genome scans identified one candidate genomic region that influenced maximum length and age‐1 to age‐2 growth. However, the limited number of candidate loci suggests that the life history traits examined may be driven by many genes of small effect or phenotypic plasticity. Although adaptive differentiation should be considered, the high inbreeding coefficients in several populations suggest that genetic rescue may benefit the most genetically depauperate WCT populations.

## 1 Introduction

Human activities are increasingly fragmenting wildlife habitat (Haddad et al. 2015), resulting in the isolation of countless, often small, populations. Such isolation puts wildlife populations at risk of extinction owing to both altered demographic and genetic processes (Reed 2004). The inevitable increase of consanguineous mating that occurs in small populations can result in stronger inbreeding depression. Inbreeding depression commonly occurs in wild populations (Hedrick and Garcia‐Dorado 2016) and can cause a decreased population growth rate and increased extirpation risk (Saccheri et al. 1998; Bozzuto et al. 2019).

Facilitating gene flow into small, isolated populations via translocations is increasingly viewed as a critical tool to address the effects of inbreeding depression (Whiteley et al. 2015; Ralls et al. 2018). Genetic rescue has been documented in a broad and increasing array of animal taxa (e.g., Westemeier et al. 1998; Madsen et al. 1999; Johnson et al. 2010; Robinson et al. 2017). Despite documented success, genetic rescue remains underused (Fitzpatrick et al. 2023). A major concern is that translocations may incidentally result in outbreeding depression by reducing important local adaptations (Edmands 2007; Bell et al. 2019). Genetic rescue efforts thus need to consider potential adaptive divergence between source and recipient populations. Adaptive differentiation in life history traits (e.g., growth and size at maturity) may be a particularly important consideration since these traits are tightly linked to fitness and, therefore, have a large influence on population growth rates (Stearns 2000).

Genomic methods that enable non‐model species to be genotyped at thousands to millions of SNPs provide valuable insights into the trade‐off between reducing inbreeding depression and potentially disrupting local adaptations (Luikart et al. 2003; Fitzpatrick and Funk 2021). For example, genomic runs of homozygosity can provide more precise estimates of inbreeding coefficients, and their use has led to studies reporting stronger effects of inbreeding depression compared to past methods (Kardos et al. 2015, 2016). Additionally, genome scans can identify loci underpinning local adaptation in key traits, adding support for a genetic influence on phenotypic variation and adaptive divergence among populations (Deagle et al. 2012; Wang et al. 2022). The risk of outbreeding depression can be reduced by minimizing divergence at these putatively adaptive loci (Fitzpatrick and Funk 2021). However, studies that use genomics to concurrently examine both local adaptation and the potential risks from inbreeding remain rare.

Salmonids are globally distributed and have high economic importance, but many populations and species are at risk from multiple stressors (Muhlfeld et al. 2018), including habitat fragmentation. Like many stream taxa, salmonids that spawn in headwaters often have highly fragmented distributions owing to the dendritic nature of stream networks (Fagan 2002), and isolated (resident) populations often have reduced genetic variation and effective population sizes (Whiteley et al. 2013; Kovach et al. 2022). In addition, salmonids often have fine‐scale local adaptations displayed through variation in life history traits such as somatic growth, size at maturity, fecundity, and egg size (Taylor 1991; Fraser et al. 2011). As a result, the translocation of maladapted individuals could disrupt local adaptations, leading to reduced fitness via outbreeding depression in the recipient population. The prevalence of fragmentation and local adaptation, combined with conservation interest and the availability of extensive genomic resources, makes salmonids a powerful and important taxon for studying the trade‐offs of translocations for genetic rescue.

In salmonids, genomic approaches to examine aspects of these tradeoffs remain rare or nonexistent for many inland trout species. Obtaining genomic measures of inbreeding is important for identifying salmonid populations facing genetic threats, yet few studies have addressed this issue (but see Perrier et al. 2017). In contrast, more research has focused on uncovering the genomic basis of life history traits in salmonids. For example, variation in traits related to somatic growth and size at maturity (Bærum et al. 2016; Carim et al. 2017, 2021) has been associated with both environmental and genetic influences (e.g., McDermid et al. 2007; Bærum et al. 2016), highlighting the role of local adaptation (e.g., Jensen et al. 2008; Drinan et al. 2012; Andrews et al. 2023). However, our understanding of loci underlying adaptive differentiation remains limited for many life history traits. A better understanding of the genetic contribution to these traits is needed, particularly for traits strongly associated with population growth in isolated systems (Carim et al. 2017). More detailed information on inbreeding and adaptive differentiation will improve our ability to identify populations most in need of genetic rescue and source populations most suitable for translocation.

The westslope cutthroat trout (*Oncorhynchus lewisi*, Page et al. 2023; hereafter “WCT”) is an excellent organism to begin exploring the use of genomics to inform genetic rescue. WCT were historically widespread across the northern Rocky Mountains, with populations supporting both fluvial and resident life forms. Like many stream‐dwelling salmonids, the distribution of WCT has been substantially reduced due to habitat loss and interactions with invasive trout species, including competition with nonnative brook trout ( *Salvelinus fontinalis* ) and hybridization with introduced rainbow trout ( *O. mykiss* ) and Yellowstone cutthroat trout ( *O. virginalis* , now considered a distinct species from WCT; Shepard et al. 2005; Muhlfeld et al. 2014, Kovach et al. 2015; Bell, Kovach, Muhlfeld, et al. 2021). Many remaining WCT populations are completely isolated and persist as small resident populations in headwater systems (Shepard et al. 2005). Due to the threat from nonnative species, reconnecting WCT populations is typically not a viable option. Instead, managers are increasingly installing barriers to intentionally isolate WCT populations and prevent negative interactions with nonnative trout species (Fausch et al. 2009). With low genetic diversity and a lack of gene flow among populations, isolated populations may be at increased risk of extirpation as they are less able to adapt to stressful and changing conditions and could suffer from inbreeding depression (O'Grady et al. 2006; Bijlsma and Loeschcke 2012). The need for genetic rescue in WCT has been evaluated, and candidate populations have been identified based on human‐caused isolation, low genetic variation, and high genetic divergence (Kovach et al. 2022). Translocations are being increasingly considered in parts of their distribution to reduce potential inbreeding depression and bolster adaptive potential. Genomics can help to make more informed decisions for the genetic rescue of WCT and refine our understanding of trade‐offs in inbreeding and outbreeding depression.

To examine these potential tradeoffs, we genotyped WCT from 25 populations in Montana, U.S.A., that varied in life history traits, demographic histories, and their isolation status to evaluate two primary research questions: (1) How do genome‐wide genetic variation and inbreeding coefficients vary across isolated and connected populations? and (2) Are there loci that underlie life history differences among isolated populations? To address these questions, we used extensive genomic data to estimate genome‐wide genetic variation (heterozygosity) and inbreeding coefficients across isolated and connected populations. We then conducted genome scans to identify potential candidate loci influencing life history differences among populations with detailed phenotypic data.

## 2 Methods

### 2.1 Study System and Sample Collection

**FIGURE 1:**
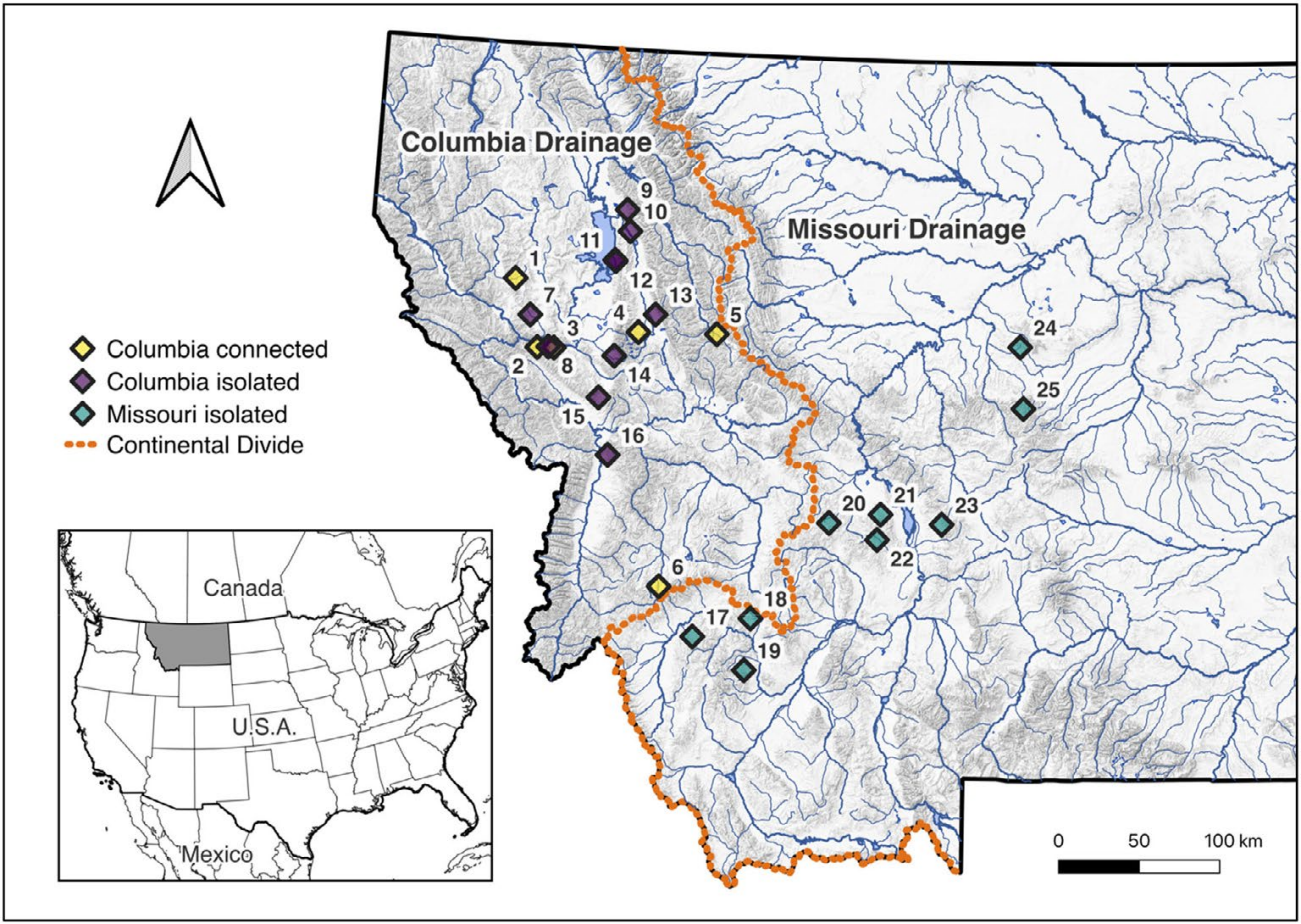
Map of the study populations categorized by drainage (Columbia or Missouri) and isolation status. Columbia isolated populations (purple) were used in the genome‐wide scans for life history related loci. Numbers correspond to population IDs, and details of populations are shown in Table 1 using these IDs.

Our study system included 25 populations spread across the Northern Rocky Mountains of Montana (Figure 1; Table 1). Eighteen of these populations reside in streams that have downstream barriers to fish passage (including geological barriers such as waterfalls and anthropogenic barriers such as perched culverts) resulting in population isolation (creating resident populations) of WCT for tens to thousands of generations. The dataset included nine populations east (Missouri River basin) and 16 populations west (Columbia River basin) of the Continental Divide of North America (Table 1). Populations in the Missouri River basin are the furthest inland of this species and underwent a historical bottleneck as WCT crossed the Continental Divide, likely during the last glacial maximum (Young et al. 2018). Data for several populations from the Missouri River basin (Staubach, North Fork Little Belt, Gold Run, and Hall) were initially collected as part of a larger study examining the fitness response of small, isolated populations to genetic rescue efforts (Bell 2022). Therefore, these populations represent a high state of inbreeding in extant WCT populations.

**TABLE 1:** Summary of study populations. Numbers correspond to population IDs in Figure 1. The sample sizes shown are for successfully genotyped fish. Demographic data are provided for populations that were included in the genome‐wide scans for life history related loci (See Carim et al. 2016, 2017 for details on life history data).

Population	# Individuals for genome scan	# Individuals for *F*_ROH_ analysis	Maximum observed length (mm)	Mean length age‐1 (mm)	Mean growth age‐1 to age‐2 (mm)
*Columbia connected*					
(1) Howell	NA	12	NA	NA	NA
(2) Seepay	NA	11	NA	NA	NA
(3) Magpie	NA	19	NA	NA	NA
(4) Red Butte	NA	23	NA	NA	NA
(5) Danaher	NA	32	NA	NA	NA
(6) Middle Fork Rock	NA	18	NA	NA	NA
*Columbia isolated*					
(7) Camas	24	12	211	62	44
(8) Magpie Spring	23	12	197	58	38
(9) Wolf	20	12	229	50	37
(10) Sixmile	25	5	215	48	39
(11) Teepee	25	13	220	58	44
(12) Talking Water	25	17	191	55	56
(13) Cooney	23	17	276	52	51
(14) Cold	25	21	256	53	41
(15) Frog	25	20	175	46	33
(16) South Fork Davis	22	6	180	48	38
*Missouri isolated*					
(17) Papoose	NA	9	NA	NA	NA
(18) South Fork North Fork Divide	NA	12	NA	NA	NA
(19) Browns	NA	12	NA	NA	NA
(20) South Fork Quartz	NA	11	NA	NA	NA
(21) Staubach	NA	12	NA	NA	NA
(22) Hall	NA	23	NA	NA	NA
(23) Ray	NA	10	NA	NA	NA
(24) North Fork Little Belt	NA	21	NA	NA	NA
(25) Gold Run	NA	11	NA	NA	NA

Westslope cutthroat trout have experienced a variety of both historic and current bottlenecks with varying influences on genomic variation. In our dataset, we expected four general demographic histories: (1) connected populations that represent the highest levels of genomic diversity with little evidence of past or current inbreeding (likely represented by connected Columbia River basin populations); (2) isolated populations with high levels of genomic variation but with evidence of recent inbreeding (likely represented by anthropogenically isolated Columbia River basin isolated populations); (3) isolated populations with low levels of genomic variation but lower levels of current inbreeding (likely represented by larger Missouri River basin isolated populations and populations that are geologically isolated); (4) isolated populations with low levels of genomic variation and high levels of inbreeding due to both historic and present bottlenecks (likely represented by smaller Missouri River basin isolated populations).

To obtain a representative genetic sample across all spawning habitat, collection of tissue was conducted at multiple locations along the length of occupied habitat in each stream (Carim et al. 2016; Bell 2022). We used information from all 18 isolated and seven connected populations to examine genome‐wide genetic variation and inbreeding coefficients (question 1). To look for loci associated with life history variation among isolated populations (question 2), we focused the analysis on the 10 isolated populations in the Columbia River basin where detailed life history information was available.

### 2.2 Genotyping and Bioinformatics

Genetic samples were prepared and sequenced according to the BestRAD protocol (a restriction associated DNA sequencing (RAD) method) for genotyping in the Montana Conservation Genomics Lab at the University of Montana in Missoula, Montana, U.S.A. We extracted DNA using an SPRI bead extraction protocol (Ali et al. 2016). DNA quality (260/280 ratio) and quantity were assessed using a Nanodrop 2000 Spectrophotometer (Thermo Scientific, Waltham MA). Samples were diluted to approximately 10 ng/μl before measuring the concentration of double‐stranded DNA using the QuantIt Picogreen assay. Double‐stranded DNA concentrations were used to standardize samples to 10 ng/μLbefore proceeding with a Pst1 BestRAD library preparation protocol (Ali et al. 2016). Individuals were labeled using BestRAD adapters with 96 unique 8‐base barcodes. NEBNext UltraII Illumina sequencing library kits (New England Biolabs, Ipswich, MA) with unique 6‐base i07 indexes were used to identify each plate. Six libraries were sequenced on 6 Illumina NovaSeq lanes using the 2 × 150 bp paired‐end read format.

Genotypes were generated following a standard pipeline which includes multiple steps to remove low‐quality data. Sequencing adapters were removed, reads were truncated whenever the mean Phred score across a window of 4 nucleotides dropped below q15, and reads were discarded if they were less than 60 bp after applying the trimming steps using Trimmomatic v0.36. Plates were demultiplexed with process_radtags, and then duplicate reads were removed using the clone_filter program from Stacks v2.55 (Rochette et al. 2019). Reads were mapped to a rainbow trout reference genome (NCBI OmykA_1.1; Gao et al. 2021) using the default setting for bwa‐mem, and resulting sam files were sorted, converted to bam format, and indexed using samtools v1.4 (Danecek et al. 2021). Genotypes were called using *gstacks* from Stacks v2.55. We used the marukilow genotyping model with ‐var‐alpha and ‐gt‐alpha set to 0.01. We then used the *populations* module to output genotypes in VCF format. Genotypes were set to missing if the genotype quality score (GQ) was less than 30 or the read depth was less than 6. Sites were removed if the minor allele was observed fewer than 3 times across all individuals, if genotypes were called in fewer than 70% of individuals, if no heterozygotes were observed, or if only heterozygotes were observed. We then used the program HDplot to identify and remove confounded paralogous loci (McKinney et al. 2017). Loci were removed if the D‐statistic calculated by HDplot was greater than 5.

### 2.3 Genotype Filtering

Many WCT contain low levels of nonnative ancestry from congeneric species that cannot be visually identified during field collection. These genomic regions of nonnative ancestry could bias estimates. To account for this, we first identified non‐admixed WCT, Yellowstone cutthroat trout, and rainbow trout using NGSadmix (Skotte et al. 2013). Three clusters (*K* = 3) cleanly split out the three species. Individuals with *Q*‐values greater than 0.99999999 were considered to be non‐hybridized. We next used ELAI (Efficient local ancestry inference) to quantify the ancestry of admixed fish, which implements a Hidden Markov model that infers the ancestry of chromosome segments continuously across the genome of individuals (Guan 2014). We ran ELAI three times with random seeds using the individuals identified as non‐hybridized as training samples. We ran ELAI with 3 upper clusters (corresponding to the three species), 15 lower clusters (5 times the number of upper clusters), and specified 10 mixing generations, as recommended by Guan (2014). We then discretized the output of each ELAI run and required that the 3 replicates yield the same dosage estimates. We created windows for every chromosome segment with contiguous ancestry. We then removed windows that were shorter than 1000 bp as an indirect way of requiring that the ancestry estimates be supported by genotypes at 2 or more RAD tags. Nonnative ancestry was generally low, with all genotyped individuals having greater than 95% WCT ancestry and the majority having no nonnative ancestry (Figure S1). Genotypes for WCT within these identified regions of nonnative ancestry were classified as missing due to the much higher than expected divergence in these regions because of the hybridization among species. This approach allowed us to exclude non‐native alleles from analyses without reducing the number of individuals analyzed. Nonnative ancestry was filtered out for the genome scan and genome‐wide heterozygosity estimates because mixed ancestry can artificially inflate estimates of heterozygosity and may result in spurious associations of loci with life history traits at a population level. In contrast, non‐native ancestry was not filtered out for detecting runs of homozygosity (described below).

We thinned to one SNP per 1 kb as an indirect way to only keep one SNP per RAD tag. We did not use additional filtering for linkage disequilibrium (Meyermans et al. 2020). We discarded loci with a missingness of 20% or greater and individuals with a missingness of 20% or greater. We used separate filtering protocols to obtain the final genotypes used in our different analyses due to differing best practices. We used a more stringent individual missingness filter of 10% for the runs of homozygosity analysis (ROH; analysis described below) because preliminary results showed strong downward bias for genomic inbreeding coefficient estimates with moderate missingness (Figure S2). The final dataset included 565 individuals in the heterozygosity analysis, 237 individuals for the genome scan (20 to 25 per population), and 371 individuals for the ROH analysis (5 to 32 per population). The mean sequencing depth for individuals used in the ROH analysis ranged from 10 to 50×, while the mean coverage for SNPs ranged from 9 to 222×.

We used a minor allele frequency (MAF) of 0.05 filter for the genome scan. However, we used a less stringent MAF filter of 0.01 for the ROH analysis because strict MAF filtering can result in downward biases in genomic inbreeding coefficient estimates (Meyermans et al. 2020). The final genotype dataset included 158,331 SNPs for inbreeding analysis and 93,323 SNPs for the genome scan.

### 2.4 Genome‐Wide Heterozygosity and Inbreeding Coefficients

We calculated mean genome‐wide expected heterozygosity to examine how genetic variation differed among populations. We generated 95% confidence intervals by bootstrapping across individuals within populations for 100 iterations. Note that we make comparisons of the mean heterozygosity in isolated versus connected populations, and in the Missouri versus Columbia drainage, but study sites were not randomly selected. Thus, these comparisons may not provide accurate estimates of differences in genetic variation among these categories. We additionally compared the number of SNPs found in the Columbia versus Missouri River drainages to examine how much variation was lost during the crossing of the continental divide. To account for having fewer sampled populations in the Missouri drainage (9), we randomly subsampled nine populations from the Columbia to quantify the number of SNPs, which was repeated 100 times. We calculated observed individual heterozygosity in non‐overlapping sliding windows of 5 Mb to examine how genetic variation was distributed across the genome.

We calculated inbreeding coefficients at the individual level to determine the severity of inbreeding within populations using the proportion of the genome in runs of homozygosity (*F*_ROH_). We detected runs of homozygosity (ROH) using a sliding window approach with the *homozyg* function in PLINK (Purcell et al. 2007; Chang et al. 2015), and a window size of 50 SNPs (‐homozyg‐window‐snp 50). We allowed one heterozygous SNP per run (‐homozyg‐het 1), which accounted for genotyping errors or rare mutations (Kardos et al. 2015). We also required a maximum missingness of 10% (5 SNPs) per window (‐homozyg‐window‐missing 5). Runs were required to have a minimum length of 5 Mb (‐homozyg‐kb 5000). As previous guidelines suggest, the minimum ROH length using 150,000 SNPs is 2 Mb (McQuillan et al. 2008), and we decided to include a minimum length of 5 Mb to ensure meaningful inference, which we had high power to detect given our SNP density of 72.6 SNPs/Mb. *F*_ROH_ was calculated as the proportion of the detectable autosomal genome that was in runs of homozygosity > 5 Mb:

$$F_{\mathrm{ROH}\left(>5\mathrm{Mb}\right)}=\frac{\sum {\mathrm{ROH}}_{>5\mathrm{Mb}}}{\text{Detectable autosomal length}}$$

The detectable genome was the sum of the length of the first SNP to the last SNP of each autosomal chromosome (Meyermans et al. 2020). We conducted ROH analyses with the same set of (158,331) SNPs for all populations because some populations were severely bottlenecked (e.g., nearly fixed homozygous chromosomes) and within‐population SNPs would not be able to detect these segments because they are identical by descent. This also allowed for direct comparison across populations while controlling for variable within‐population SNP density. We additionally calculated *F*_ROH_ with ROH > 10 Mb and ROH > 20 Mb, which had similar patterns among populations (Figure S3). We also calculated inbreeding at the population level to determine genetic differentiation among populations using Weir and Cockerham's *F*_ST_ (Weir and Cockerham 1984). We randomly selected 10,000 loci for the analysis to reduce linkage and decrease processing time. *F*_ST_ was calculated in the *R* package *Hierfstat* (Goudet 2005).

Together, these analyses can provide insight into the demographic histories of populations (Robinson et al. 2019). For example, recently bottlenecked populations should have high levels of background genetic variation but also high inbreeding coefficients (see the demographic histories described above).

### 2.5 Genome‐Wide Scans for Life History Related Loci

Genome scans only included populations in the Columbia River basin that had available life history information, which consisted entirely of isolated, resident populations (Table 1). Our traits were treated as population level covariates, and although individual level data were not available, population level comparisons are valuable for identifying outlier loci that could contribute to adaptive differentiation and potential outbreeding depression. We used three previously estimated life history traits in our genome scans: maximum observed fish length in a population, mean length of age‐1 fish, and mean change in length (i.e., somatic growth) from age‐1 to age‐2 (Carim et al. 2017). Maximum observed length corresponds to the length of the largest fish observed during population surveys (see Carim et al. 2016). Age‐1 length and age‐1 to age‐2 growth were based on back‐calculated length‐at‐age from sagittal otoliths collected from a subset of individuals surveyed in each population. Although interannual variation in the environment likely influences these traits, traits were measured over the course of multiple years, making our estimates closer to population means over approximately one generation. These traits represent key aspects of life history variation among populations, and were shown to be influential on growth rate (lambda) of these populations (Carim et al. 2017). For example, maximum observed length could be associated with differences in fecundity, iteroparity and lifespan (e.g., populations with larger fish may have higher rates of adult survival, and adults may produce more offspring over their lifespan). Age‐1 length and age‐1 to age‐2 growth are associated with investing resources to grow quickly and achieve an earlier age‐at‐maturity. Several populations in our dataset have small maximum lengths and rapid early growth (e.g., Teepee and Talking Water), likely indicating that some populations have a “live fast die young” life history strategy. These life history traits had moderate to low correlations, ranging from 0.12 to 0.41, thus allowing for genome scans to be independent.

We used *BayPass* to scan genomes for SNP loci associated with life history differences among isolated WCT populations (Gautier 2015). *BayPass* uses a Bayesian framework that accounts for correlated allele frequencies due to background genetic structure (Figure S4 & S5), identifies SNPs that are overly differentiated based on the XtX statistic, and tests for associations between SNPs and population‐specific variables (Gautier 2015; Olazcuaga et al. 2021). The XtX statistic can be thought of as a SNP‐specific *F*_ST_ that accounts for the covariance in population allele frequencies (Gautier 2015). We used the standard model with a neutral covariance matrix. The XtX significance is based on a 1% probability for a simulated pseudo‐observed dataset (≥ 20). Although we focused on finding outliers for life history traits, we also report the total number of XtX outliers at a more stringent 0.01% probability for a simulated pseudo‐observed dataset (≥ 30), since these outliers could underlie other traits with adaptive divergence. Support for loci underlying life history differentiation was quantified using Bayes factors measured in deciban units (dB; 10 log_10_ Bayes Factor), with a Bayes factor ≥ 20 dB being considered decisive evidence that a locus is an outlier (Jefferys 1961). Loci that are outliers for both XtX and Bayes factors associated with a trait are considered candidate loci that could underlie adaptive divergence in life history traits. Candidate loci were mapped to a rainbow trout reference genome (NCBI OmykA_1.1) and genes with candidate SNPs were recorded. We additionally calculated Pearson's correlation between population allele frequencies and traits as a secondary test of associations for the candidate loci, one that does not correct for differences in underlying population genetic structure.

## 3 Results

### 3.1 Genetic Variation and Inbreeding

**FIGURE 2:**
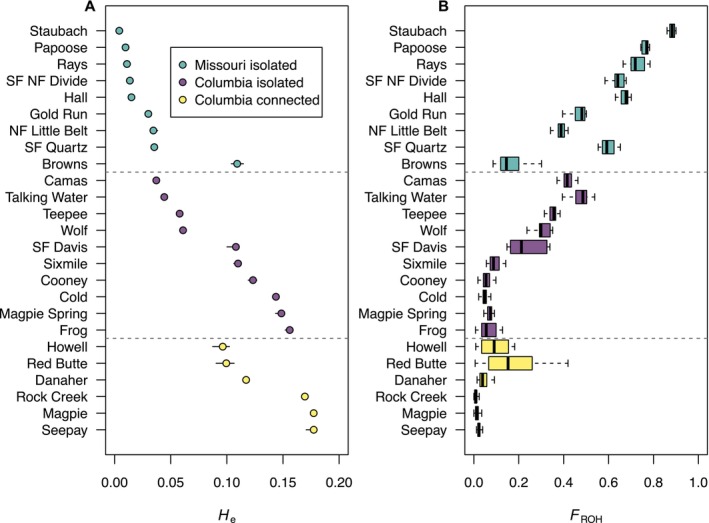
Genome‐wide expected heterozygosity (*H*_e_; A) and genomic inbreeding coefficients (*F*_ROH_; B) estimates. Confidence intervals (95%) in A were generated by bootstrapping across individuals.

Genome‐wide heterozygosity (*H*_e_) varied considerably among populations throughout the range of WCT in Montana (Figure 2a). The population with the highest genetic variation (*H*_e_ = 0.183) had 43 times greater heterozygosity than the lowest (*H*_e_ = 0.004). Connected populations in the Columbia River basin typically had the highest genetic variation (median *H*_e_ = 0.146). Isolated populations in the Columbia River basin had slightly lower heterozygosity on average (median *H*_e_ = 0.112). Isolated populations in the Missouri River basin had considerably lower genetic variation (median *H*_e_ = 0.015). However, Browns Creek had a substantially higher *H*_e_ (0.11) compared to other isolates in the Missouri River basin. WCT in the Columbia drainage had 1.7 times more SNPs (140,617, 95% CI = 135,656 to 142,820) than the Missouri River drainage (80,844). Pairwise *F*_ST_ ranged from 0.046 to 0.872, with the small isolated populations generally having the greatest *F*_ST_ estimates (Table S1). *F*_ST_ was also high for comparisons between populations in the Columbia and Missouri drainages.

**FIGURE 3:**
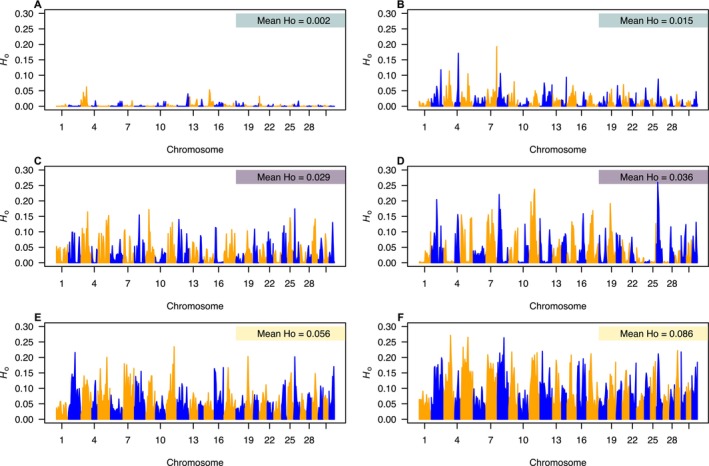
Genome‐wide observed heterozygosity for a sample of six selected individuals from populations with differing demographic histories. Populations include isolated Missouri populations (A) Staubach and (B) North Fork Little Belt, isolated Columbia populations (C) Teepee and (D) South Fork Davis, and connected Columbia populations (E) Danaher and (F) Seepay. The genome‐wide observed heterozygosity (Mean *H*_o_) is highlighted in colored boxes.

Consistent with genome‐wide heterozygosity, genomic inbreeding coefficients (*F*_ROH > 5Mb_) varied considerably among individuals, ranging from 0 to 0.90 (Figure 2b). Connected populations in the Columbia River basin had the lowest *F*_ROH_ (median = 0.028), followed by isolated populations in the Columbia River basin (median = 0.114), and the Missouri River basin, which had the highest *F*_ROH_ (median = 0.64; Figure 2b). Some populations in the Missouri River basin had extreme levels of inbreeding. For example, individuals in Staubach Creek had a mean *F*_ROH_ of 0.882. The populations with the highest inbreeding have multiple chromosomes where all loci are entirely in runs of homozygosity (Figure 3a). For example, in Staubach Creek, several chromosomes (e.g., chromosomes 5, 9, 27–32) appear to be entirely in runs of homozygosity (i.e., *H*_o_ = 0) for all individuals.

The distribution of heterozygosity across the genome further highlights the differing demographic histories experienced by WCT in Montana. First, many populations from the Missouri River basin showed evidence for both past and current bottlenecks (e.g., Staubach; Figure 3a). Second, several populations in the Missouri River basin appear to have low genome‐wide variation but relatively low levels of recent inbreeding (e.g., North Fork Little Belt; Figure 3b). Third, particularly for some isolated populations in the Columbia River basin, we observed high rates of recent inbreeding, although relatively high levels of genome‐wide heterozygosity were maintained compared to most populations in the Missouri River basin (e.g., Teepee and South Fork Davis Figure 3c,d). Finally, many connected populations in the Columbia River basin, and some larger isolated populations, have relatively high genome‐wide variation and minimal recent inbreeding, suggesting the populations have remained at a large size for many generations (e.g., Danaher and Seepay, Figure 3e,f).

### 3.2 Adaptive Differentiation in Life History Traits

**FIGURE 4:**
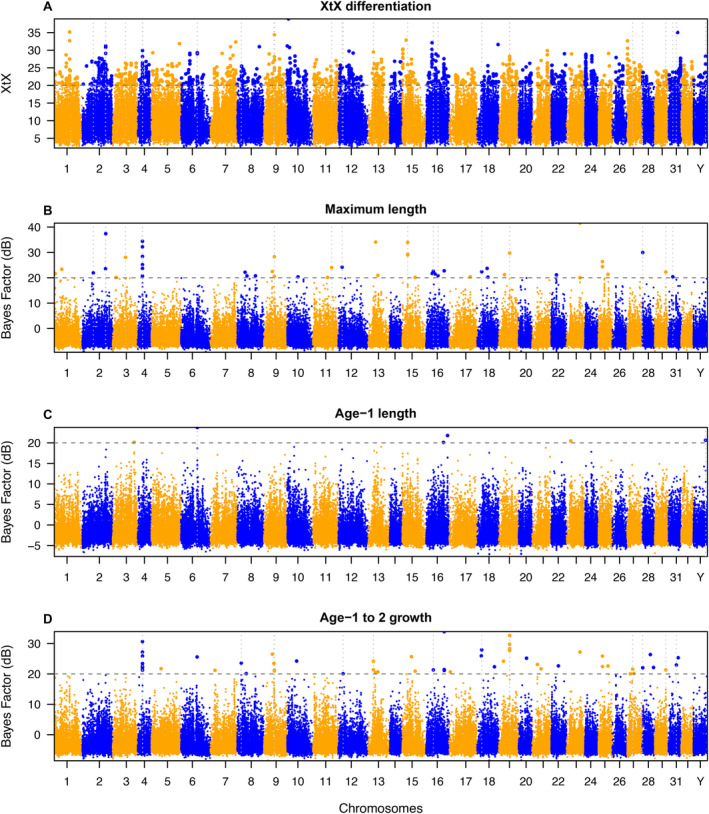
Genome‐wide scan for SNPs showing adaptive differentiation among isolated populations in the Columbia drainage, including (A) XtX differentiation and Bayes factors for associations with (B) maximum observed length, (C) mean age‐1 length, and (D) mean age‐1 to age‐2 growth. Vertical dashed lines represent loci that are significant outliers for both XtX and a Bayes factor associated with that trait. Blue and orange represent chromosome breaks. The horizontal dashed line represents significance thresholds.

We detected 36 candidate outlier SNPs that were greater than both the XtX (20) and the Bayes factor (20 dB) thresholds for at least one life history associated trait, with 28 of these candidate loci falling within genes. Thirteen SNPs were significant for more than one of the three studied life history traits, resulting in a total of 49 significant associations with these traits. Candidate outliers were detected for all three life history traits (Figure 4), including two candidate SNPs underlying age‐1 length, 22 SNPs underlying maximum observed length, and 25 SNPs underlying age‐1 to age‐2 growth (Table S2).

The genome region with the strongest support was between 16,156 and 16,272 kb on chromosome 4, which had eight candidate outliers, six of which were associated with both maximum length and age‐1 to age‐2 growth. Additionally, three of the six Bayes factors over 30 dB (considered extremely strong evidence of an outlier) were in this region, as was the candidate locus with the highest XtX value. Six of these outliers aligned to the *nkain2* gene. However, the outlier loci in this region had only moderate Pearson's correlations between population allele frequencies and trait values (Table S2), and the locus with the highest Bayes factor had a correlation of 0.67 (Figure S6).

We detected an additional 21 candidate outliers that did not meet the Bayes factor threshold but did meet the more stringent XtX threshold (30). Of these, ten mapped to genes (Table S3). No SNPs were above both the Bayes factor threshold and the more stringent XtX threshold. This suggests that additional outlier loci may be associated with traits that we did not measure.

## 4 Discussion

Using over 150,000 SNPs, we show that the smallest isolated WCT populations have extremely low genome‐wide genetic variation and high inbreeding coefficients. Despite considerable trait variation among isolated populations, we found only moderate evidence for candidate loci underpinning life history traits. Although outbreeding depression could still be a concern, these data provide further evidence that assisted translocations for genetic rescue are warranted in some cases for WCT and likely many other fish species that are similarly fragmented.

The trends in genome‐wide genetic variation in WCT are consistent with past studies that used fewer than 100 loci (Leary et al. 1988; Drinan et al. 2011; Kovach et al. 2022), with isolated populations and populations in the Missouri River basin showing substantially reduced genetic variation. With high‐resolution genomic data, we show the severity of the loss of genetic variation in bottlenecked and isolated populations in detail. In the smallest populations, many individuals have multiple chromosomes where loci appear to be nearly or entirely contained in runs of homozygosity. We also report that WCT in Montana has levels of inbreeding that are similar to or even greater than those documented in severely bottlenecked wildlife populations, including wolves (Kardos et al. 2018), ibex (Grossen et al. 2020), and tigers (Khan et al. 2021). Despite challenges with comparing inbreeding across taxa, the severe loss of genetic variation we report undoubtedly shows that some WCT populations are highly inbred.

The low genomic variation and high degree of inbreeding found in the smallest WCT populations can reduce individual fitness (e.g., inbreeding depression; Bell et al. 2024), potentially increasing extirpation risk. More generally, the smallest WCT populations in our study had similar levels of inbreeding to Isle Royale wolves (Robinson et al. 2019), and inbreeding depression contributed to the functional extinction of that wolf population (Hedrick et al. 2019). Several recent studies using simulations have suggested that purging of deleterious alleles can reduce or nullify the risks of inbreeding depression in small, isolated populations (Kyriazis et al. 2021; Robinson et al. 2022). However, purging is less likely to occur in recently bottlenecked populations (i.e., tens of generations). Further, strong empirical evidence suggests that a high genetic load (i.e., lower average fitness due to the presence of deleterious alleles in a population) is often maintained in small populations (Johnson et al. 2010; Adams et al. 2011; Bozzuto et al. 2019), and it is likely only the largest effect loci that are purged (Grossen et al. 2020). In addition to the immediate threats from inbreeding depression and high genetic load, low genomic variation can also hamper a population's ability to adapt to future change in the environment (Mathur et al. 2023). These threats are highly relevant for WCT in Montana, where many populations are as small as those examined here (Kovach et al. 2022).

Despite the known risks associated with population isolation, practitioners are increasingly relying on intentional isolation for native species management (Rahel 2013), which may become more necessary as climate change facilitates invasive species expansion into remaining WCT populations (Muhlfeld et al. 2014). Multiple populations in our study (Browns, Staubach, Ray, Red Butte, Seepay, and Magpie) were intentionally isolated to prevent the spread of nonnative species into these systems. Our estimates of inbreeding suggest some isolated populations may be large enough to negate the risks of inbreeding depression. With low levels of inbreeding and high genetic variation, it is possible that populations like Browns and Seepay may be large enough to maintain genetic variation unless impacted by a significant environmental event (Peterson et al. 2014). However, many of the other isolated populations already display reduced genetic variation and increased inbreeding, emphasizing that intentional isolation—though necessary—comes at a clear genetic cost. Fortunately, the genetic costs may be remedied with genetic rescue.

In many ways, genetic rescue seems like an ideal tool for WCT genetic management, but its implementation may be complex (Tallmon et al. 2004). Decades of research suggest that outbreeding depression may be particularly concerning for salmonids due to strong evidence for local adaptation (Taylor 1991; Fraser et al. 2011), including from genomic studies. Several studies have found large effect genes that underlie key components of life history variation linked to local adaptation, primarily migration timing (Prince et al. 2017), age at maturity (Ayllon et al. 2015), and migration tendency (Pearse et al. 2019). Despite previous work demonstrating evidence for local adaptation in WCT (Drinan et al. 2012; Carim et al. 2017), we did not find strong evidence for loci underlying life history differences among these populations. Here, the candidate SNPs with the strongest support were within the *nkain2* gene. *Nkain2* is a sodium/potassium transporting ATPase and has been associated with height, weight, and body mass index in humans (NHGRI GWAS Catalog). However, the statistical support for this association was relatively weak, and this gene has not been reported in other studies that conducted genome scans on salmonid life history traits (e.g., Barson et al. 2015). It is worth noting that we observed additional outliers that were not associated with the traits of interest, with the strongest outlier mapping to the *trpc6a* gene. This gene, along with others in the transient receptor channel gene family, has been associated with sensing thermal conditions in marine fishes (York and Zakon 2022; Huang et al. 2024). Further study into how these genes influence traits, particularly in cold‐water salmonids, could provide information on potential drivers of local adaptation among the populations in our dataset.

Although we did not find an obvious genetic basis for the few aspects of WCT life history differences we examined, our study had several limitations. Specifically, we had a low sample size of ten populations with phenotypic data, the populations in our dataset exhibit complex genetic structure and strong genetic drift, and we had limited phenotypic information (i.e., no individual level data were available for genome scans). Further, some of our populations had low levels of hybridization with rainbow trout. Although nonnative ancestry from rainbow trout was removed prior to the analysis to meet assumptions concerning genetic structure for the genome scan, rainbow trout hybridization could have contributed to observed differences in life history traits. Given that we only had power to detect large effect loci (Hoban et al. 2016), many small to medium effect loci could also be responsible for adaptive differentiation (e.g., Sinclair‐Waters et al. 2020). Overall, the results presented in this effort should be taken as an initial investigation into the genetic basis of local adaptation for life history traits in WCT; much work remains. More generally, local adaptation and outbreeding depression should continue to be considered in any discussions concerning translocations of salmonid fishes for purposes of genetic rescue. That being said, in small isolated populations genetic drift overwhelms natural selection (Wright 1931), which may suggest that local adaptation may be least likely in those populations where, in the context of genetic rescue, we are most concerned about its presence.

The challenge for WCT and salmonid fishes more generally is to identify candidate populations that are most likely to benefit from rescue, and donor populations that are least likely to inadvertently cause outbreeding depression while ideally maximizing heterosis. Generally speaking, pre‐existing genetic baselines that already exist for many salmonid species are likely sufficient for identifying candidates for genetic rescue (e.g., Kovach et al. 2022). Genomic data simply provide a high‐resolution means to confirm that lower genetic variation measured at smaller sets of genetic markers in some populations does indeed reflect higher inbreeding and lower genome‐wide diversity. However, genetic monitoring programs are not ubiquitous for all salmonid fishes and are even rarer outside of salmonid fishes. Genomic estimates of *F*_ROH_ are likely to be particularly useful for species without extensive genetic baselines, where candidate populations for rescue might be identified solely based on ecological criteria (isolation and small population size), with genomic data used to confirm that inbreeding is indeed a concern. For example, genetic rescue might be deemed warranted if *F*_ROH_ is greater than a predefined threshold. However, replicated empirical evidence, possibly taxon‐specific, will likely be necessary to determine such *F*_ROH_ thresholds. Another strength of genomic methods lies in their ability to help identify the source populations (or even individuals) most suitable for genetic rescue translocations. For example, selecting individuals with patterns of ROH that differ from those of the recipient population may be an effective way to reduce inbreeding (e.g., Bossu et al. 2023), even if all potential source populations are themselves inbred.

Using genomics to predict when outbreeding depression may occur is more difficult, especially for species like WCT where the genetic architecture of local adaptation, if it exists, remains unknown and is likely variable within and among populations (e.g., Therkildsen et al. 2019). For example, even some of the genes of large effect that have been identified in salmonid fishes have regionally varying effects on phenotypic variation (e.g., Narum et al. 2024). Given this, gene‐specific guidance for genetic rescue is challenging (Kardos and Shafer 2018). Outlier loci can be used as a complementary, but not the primary, source of information to help optimize source population selection when possible. Conservation efforts with access to genomic data can aim to minimize divergence in outlier loci. Special attention can be given to outlier loci associated with traits that directly influence fitness and population growth, such as life history traits, since these loci may be more likely to contribute to outbreeding depression that appreciably influences population dynamics (Bell, Kovach, Robinson, et al. 2021). However, additional outlier loci that are not associated with measured traits can also provide information about potential adaptive differentiation. For example, the strongest outlier loci in our analyses were not associated with life history traits. Our study examined broader patterns in inbreeding and adaptive divergence of isolated WCT populations, rather than specifically identifying potential source populations for genetic rescue. However, our findings could inform future work aimed at identifying adaptive loci to aid in genetic rescue implementation (Fitzpatrick and Funk 2021).

In our opinion, genomics should be seen as an additional source of information that can be helpful, but not essential, to reduce outbreeding depression risks. General rules of thumb that have been previously published are likely most robust and broadly applicable (Frankham et al. 2017; Fitzpatrick and Funk 2021). In short, sources for genetic rescue actions should occur in populations with similar environmental habitats and be historically connected prior to recent human‐induced fragmentation (Frankham et al. 2011). In the case of WCT, this immediately excludes assisted translocation actions between populations in the Missouri and Columbia River basins, as they have been evolutionarily isolated for tens of thousands of years and are arguably distinct evolutionary units (Young et al. 2018). If a population is at severe risk from inbreeding, higher‐risk translocations can be considered since genetic rescue can still occur using adaptively divergent source populations (Fitzpatrick et al. 2016; Fitzpatrick and Funk 2021). Refinement of guidelines to reduce the risk of outbreeding depression will require further empirical investigation into genetic rescue outcomes in salmonid populations.

Taken together, our results provide further evidence that the potential genetic benefits of human‐facilitated gene flow likely outweigh the risks of outbreeding depression for the smallest WCT populations isolated by human activities (Kovach et al. 2022). The low genetic variation and high divergence of WCT demonstrate the potential for gene flow to greatly reduce inbreeding and increase genetic variation, likely leading to reduced inbreeding depression and increased adaptive variation. Genetic “flatlining” of the smallest populations highlights the stronger influence of genetic drift relative to selection, but given the nature of genetic drift, many of these populations still contain unique variation that warrants protections (Allendorf and Leary 1988; Kovach et al. 2022). Gene flow could thus result in the genetic “resuscitation” of these populations. Genetic rescue attempts in at‐risk populations have been very limited in fishes (Frankham et al. 2017), but attempts are increasing (Fitzpatrick et al. 2016; Robinson et al. 2017; Kronenberger et al. 2018; Bell 2022; Pregler et al. 2023), and this management action is increasingly incorporated into conservation strategies. For example, genetic rescue is now included in the conservation plan for WCT in southwestern Montana (Jaeger et al. 2022). As such, the next decade(s) will likely reveal much about the risks of inbreeding and outbreeding depression in salmonid fishes.

## Conflicts of Interest

The authors declare no conflicts of interest.

## Supporting information


Data S1.

## Data Availability

Data for this study are available at the Dryad Digital Repository: https://doi.org/10.5061/dryad.6hdr7srbm.
