# Supplementary material for: Genomic Insights Into Inbreeding and Adaptive Divergence of Trout Populations to Inform Genetic Rescue
Source: Evol Appl. 2025 Mar 20;18(3):e70090. doi: 10.1111/eva.70090 (PMC11923392; doi:10.1111/eva.70090)
Supplement: Supplementary file 1 — Data S1. [file EVA-18-e70090-s001.pdf]

Supplementary Materials

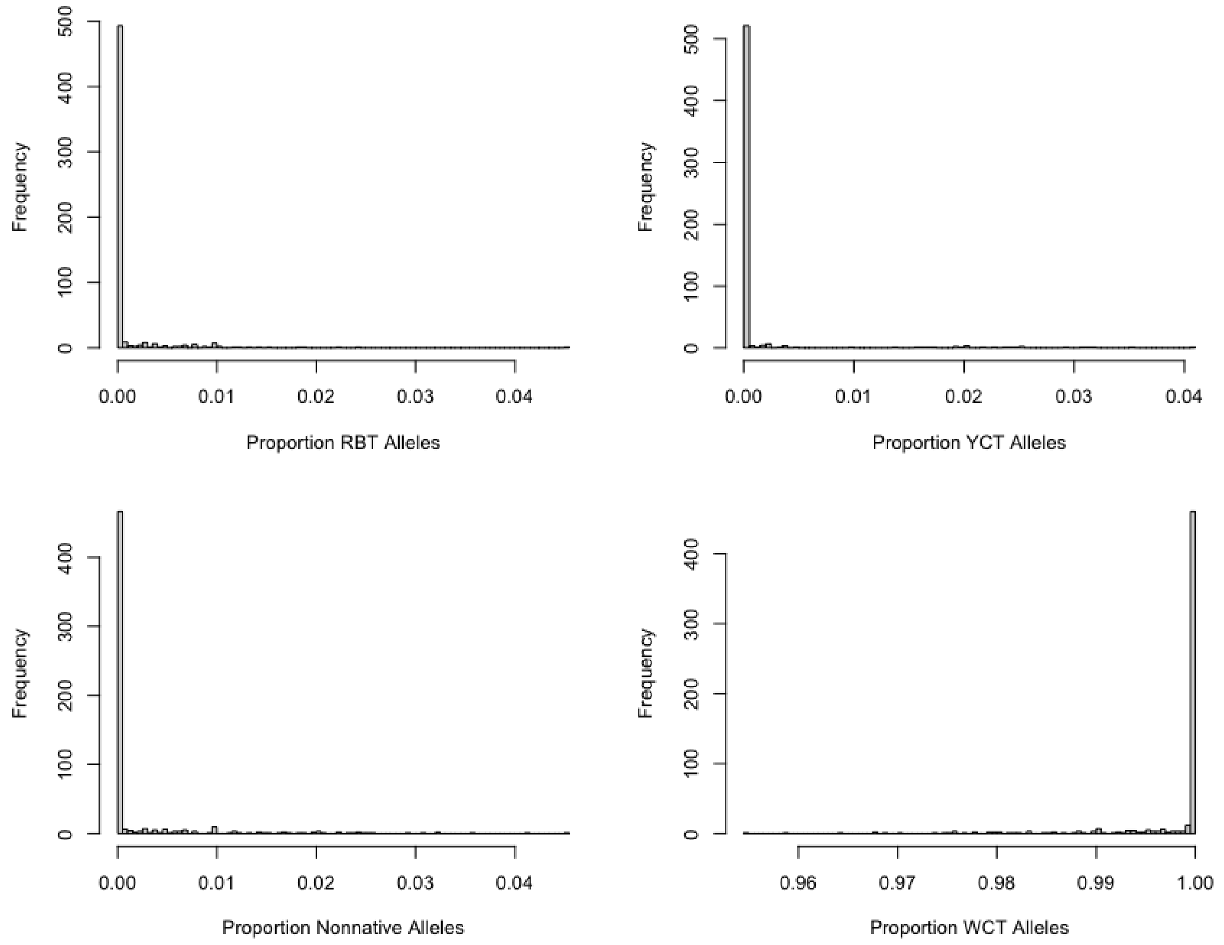

Fig. S1. Histograms of the proportion of nonnative (Rainbow trout (RBT), Yellowstone cutthroat trout (YCT), and total nonnative) and native (Westslope cutthroat (WCT)) alleles for all individuals included in analyses. All individuals had under less than 5% nonnative ancestry and the majority had no nonnative ancestry.

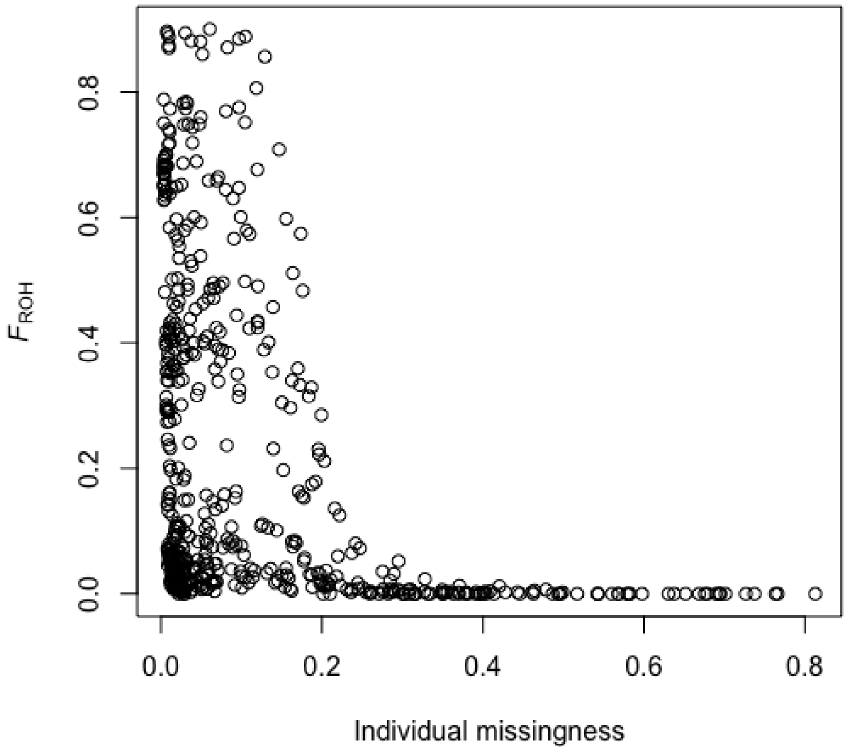

Fig. S2. Genomic inbreeding estimates ($F_{ROH(>5\ Mb)}$) versus individual missingness. High individual missingness causes a strong downward bias of F_{ROH}.

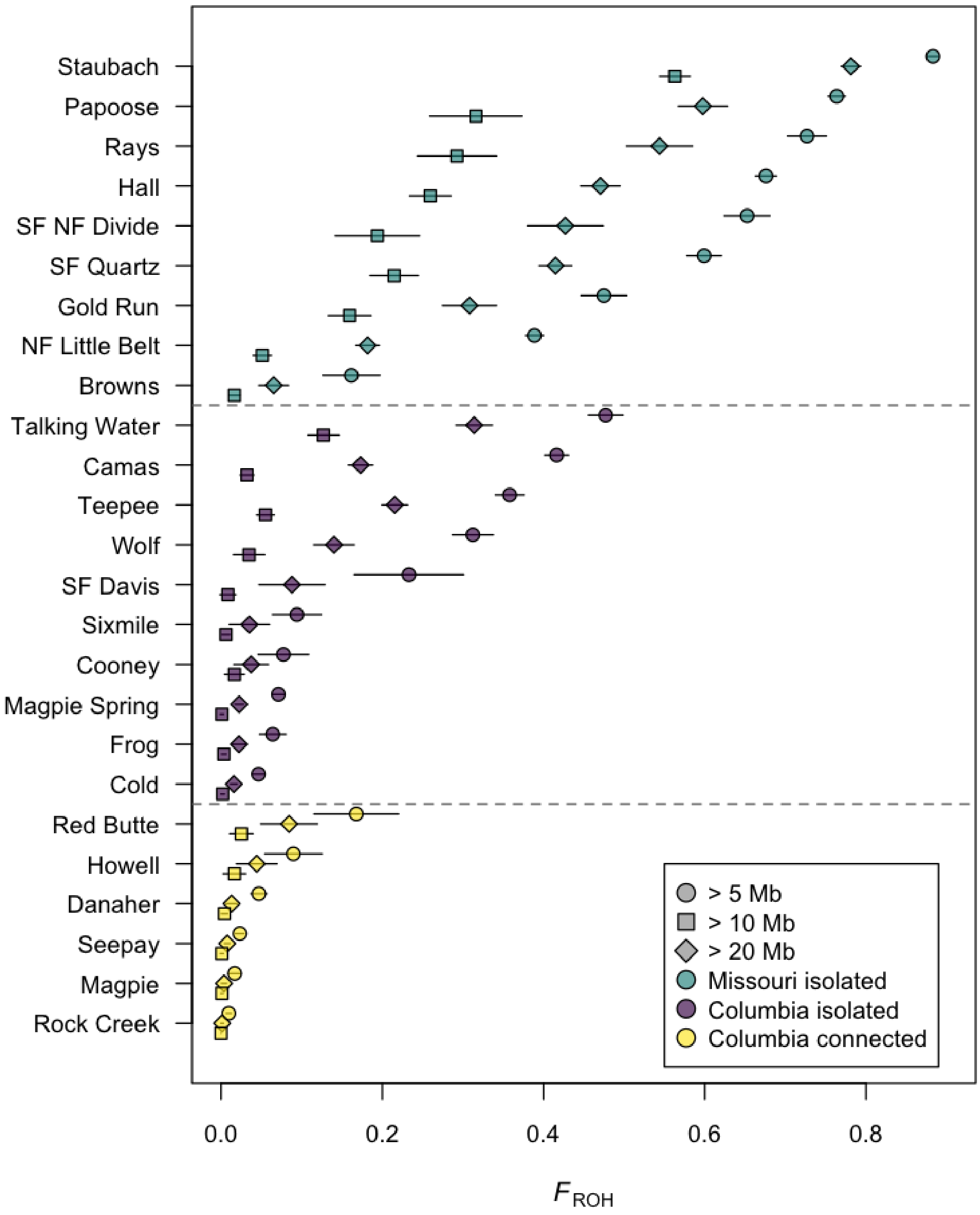

Fig. S3. Estimates of F_{ROH} with different minimum run of homozygosity lengths, including 5 Mb (used in manuscript), 10 Mb, and 20 Mb. Points are population means with 95% confidence bands.

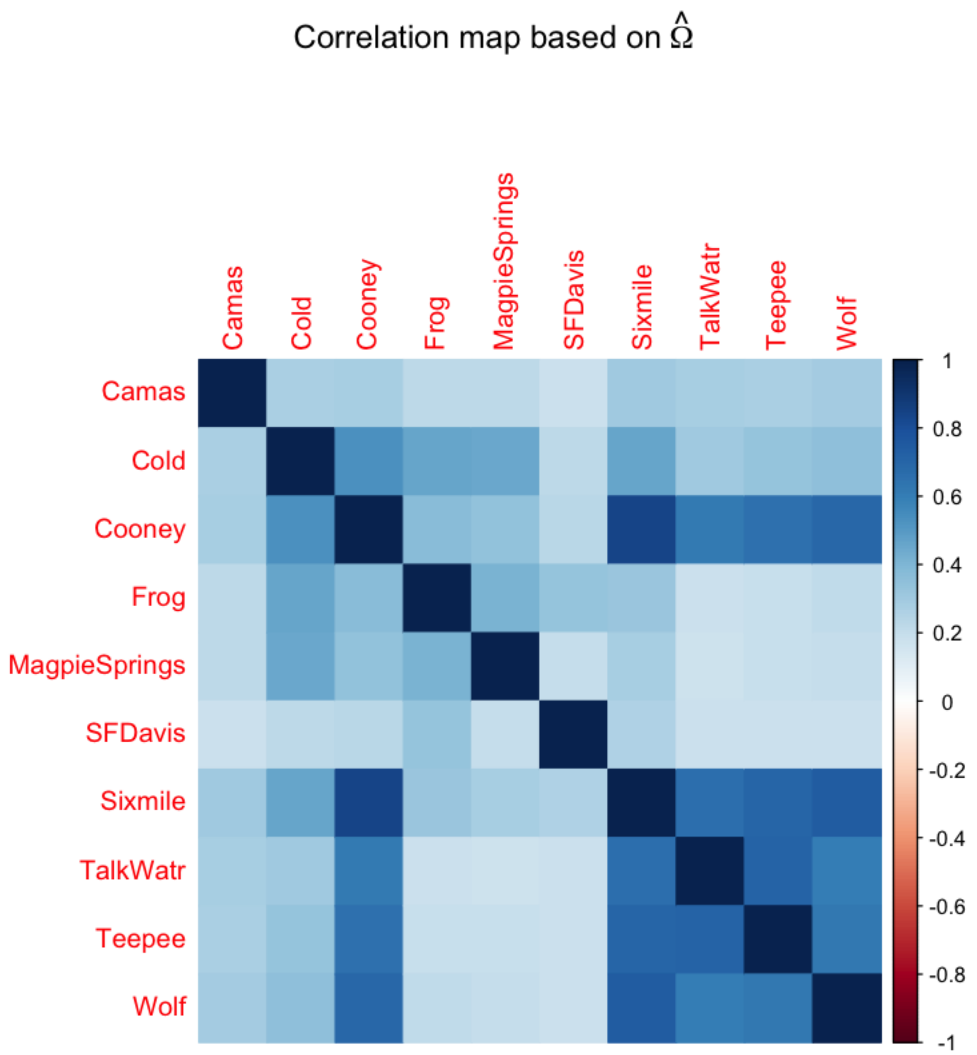

Fig. S4. Correlation plot of the covariance matrix **Ω** of population allele frequencies using isolated populations in the Columbia drainage, produced from the BayPass analysis.

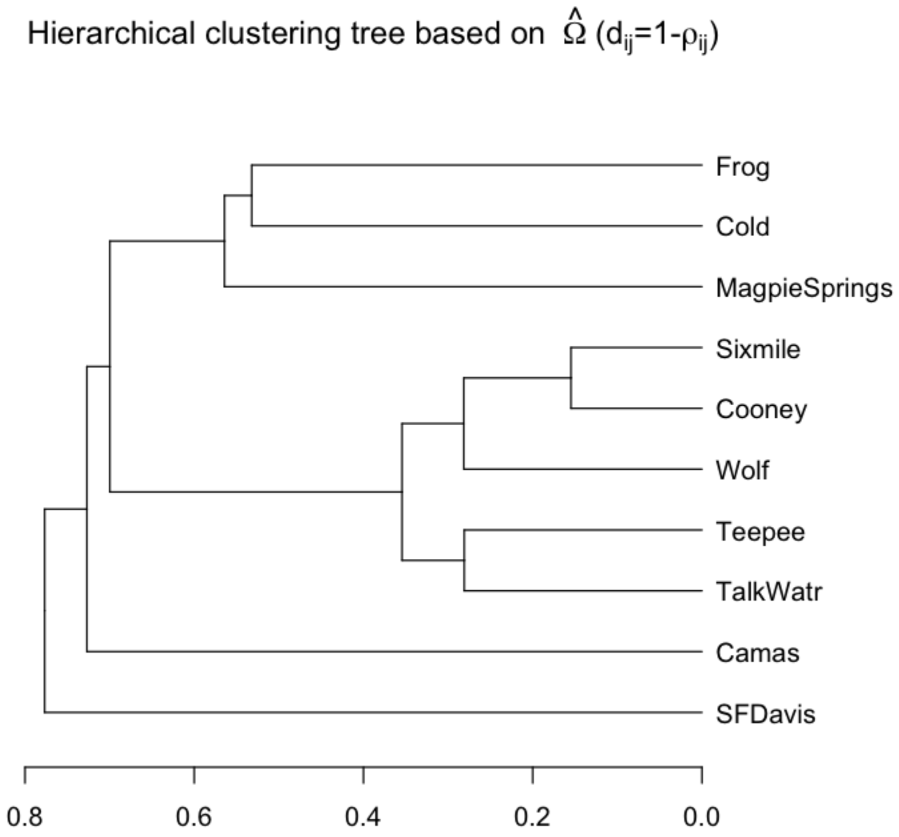

Fig. S5. Hierarchical clustering tree based on the covariance matrix **Ω** of allele frequencies among populations using isolated populations in the Columbia drainage, produced from the BayPass analysis.

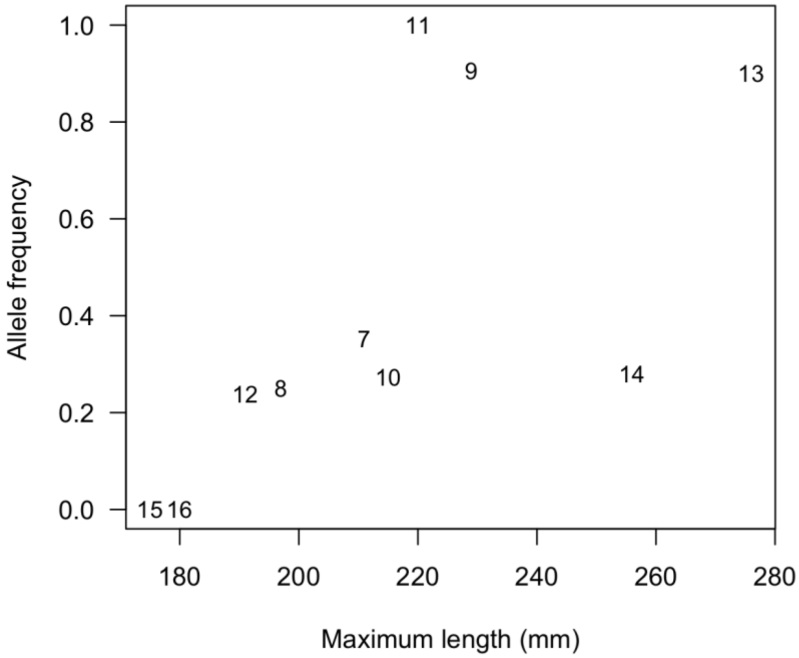

Fig. S6. Maximum observed length (mm) versus allele frequency at the locus with the strongest support in the outlier test (Chromosome 4, 16,174,986 base pair). The Pearson's correlation was equal to 0.67. Numbers correspond to population IDs in Table 1 and Figure 1 in the main text.

Table S1. Pairwise Weir and Cockerham's F_{ST} among the 25 study populations. Colors scale with F_{ST} estimates, with green representing lower values and red representing higher values.

	Browns	Camas	Cold	Cooney	Danaher	Davis	Divide	Frog	Gold Run	Hall	Howell	Little Belt	Magpie	Magpie Springs	Papoose	Quartz	Rays	Red Butte	Rock Creek	Seepay	Sixmile	Staubach	Talking Water	Teepee	Wolf
Browns		0.57	0.18	0.17	0.24	0.40	0.28	0.21	0.29	0.32	0.25	0.32	0.16	0.25	0.30	0.30	0.29	0.26	0.21	0.19	0.18	0.33	0.48	0.42	0.38
Camas	0.57		0.48	0.52	0.53	0.64	0.85	0.47	0.79	0.85	0.62	0.79	0.42	0.51	0.85	0.78	0.85	0.59	0.46	0.45	0.56	0.87	0.78	0.73	0.71
Cold	0.18	0.48		0.15	0.23	0.35	0.46	0.16	0.40	0.49	0.23	0.44	0.09	0.19	0.46	0.41	0.46	0.24	0.18	0.13	0.17	0.48	0.41	0.36	0.33
Cooney	0.17	0.52	0.15		0.18	0.37	0.45	0.18	0.37	0.49	0.13	0.42	0.12	0.22	0.45	0.40	0.45	0.12	0.19	0.15	0.06	0.48	0.36	0.30	0.25
Danaher	0.24	0.53	0.23	0.18		0.39	0.46	0.23	0.40	0.49	0.25	0.43	0.20	0.29	0.46	0.42	0.46	0.27	0.20	0.23	0.18	0.48	0.44	0.39	0.36
Davis	0.40	0.64	0.35	0.37	0.39		0.66	0.31	0.60	0.70	0.44	0.64	0.28	0.38	0.67	0.59	0.67	0.45	0.25	0.30	0.39	0.69	0.62	0.57	0.55
Divide	0.28	0.85	0.46	0.45	0.46	0.66		0.45	0.60	0.42	0.56	0.56	0.41	0.52	0.53	0.57	0.48	0.53	0.45	0.46	0.48	0.61	0.78	0.72	0.71
Frog	0.21	0.47	0.16	0.18	0.23	0.31	0.45		0.39	0.49	0.25	0.44	0.11	0.20	0.45	0.40	0.45	0.26	0.15	0.13	0.20	0.47	0.42	0.37	0.35
Gold Run	0.29	0.79	0.40	0.37	0.40	0.60	0.60	0.39		0.61	0.47	0.43	0.35	0.46	0.63	0.57	0.61	0.45	0.39	0.40	0.39	0.69	0.71	0.65	0.63
Hall	0.32	0.85	0.49	0.49	0.49	0.70	0.42	0.49	0.61		0.59	0.56	0.44	0.56	0.49	0.58	0.47	0.56	0.49	0.51	0.51	0.60	0.79	0.74	0.73
Howell	0.25	0.62	0.23	0.13	0.25	0.44	0.56	0.25	0.47	0.59		0.52	0.19	0.29	0.56	0.50	0.56	0.22	0.25	0.23	0.14	0.59	0.46	0.39	0.35
Little Belt	0.32	0.79	0.44	0.42	0.43	0.64	0.56	0.44	0.43	0.56	0.52		0.40	0.51	0.57	0.56	0.57	0.50	0.44	0.45	0.44	0.62	0.72	0.67	0.65
Magpie	0.16	0.42	0.09	0.12	0.20	0.28	0.41	0.11	0.35	0.44	0.19	0.40		0.12	0.40	0.36	0.41	0.20	0.13	0.05	0.14	0.43	0.37	0.32	0.29
Magpie Springs	0.25	0.51	0.19	0.22	0.29	0.38	0.52	0.20	0.46	0.56	0.29	0.51	0.12		0.52	0.46	0.52	0.30	0.21	0.13	0.24	0.54	0.48	0.42	0.39
Papoose	0.30	0.85	0.46	0.45	0.46	0.67	0.53	0.45	0.63	0.49	0.56	0.57	0.40	0.52		0.60	0.57	0.53	0.45	0.46	0.48	0.73	0.78	0.73	0.71
Quartz	0.30	0.78	0.41	0.40	0.42	0.59	0.57	0.40	0.57	0.58	0.50	0.56	0.36	0.46	0.60		0.57	0.49	0.39	0.40	0.42	0.65	0.72	0.66	0.64
Rays	0.29	0.85	0.46	0.45	0.46	0.67	0.48	0.45	0.61	0.47	0.56	0.57	0.41	0.52	0.57	0.57		0.53	0.45	0.47	0.48	0.65	0.78	0.73	0.71
Red Butte	0.26	0.59	0.24	0.12	0.27	0.45	0.53	0.26	0.45	0.56	0.22	0.50	0.20	0.30	0.53	0.49	0.53		0.27	0.24	0.14	0.56	0.44	0.37	0.33
Rock Creek	0.21	0.46	0.18	0.19	0.20	0.25	0.45	0.15	0.39	0.49	0.25	0.44	0.13	0.21	0.45	0.39	0.45	0.27		0.14	0.20	0.47	0.43	0.38	0.35
Seepay	0.19	0.45	0.13	0.15	0.23	0.30	0.46	0.13	0.40	0.51	0.23	0.45	0.05	0.13	0.46	0.40	0.47	0.24	0.14		0.18	0.48	0.42	0.37	0.33
Sixmile	0.18	0.56	0.17	0.06	0.18	0.39	0.48	0.20	0.39	0.51	0.14	0.44	0.14	0.24	0.48	0.42	0.48	0.14	0.20	0.18		0.51	0.39	0.32	0.27
Staubach	0.33	0.87	0.48	0.48	0.48	0.69	0.61	0.47	0.69	0.60	0.59	0.62	0.43	0.54	0.73	0.65	0.65	0.56	0.47	0.48	0.51		0.81	0.75	0.74
Talking Water	0.48	0.78	0.41	0.36	0.44	0.62	0.78	0.42	0.71	0.79	0.46	0.72	0.37	0.48	0.78	0.72	0.78	0.44	0.43	0.42	0.39	0.81		0.57	0.59
Teepee	0.42	0.73	0.36	0.30	0.39	0.57	0.72	0.37	0.65	0.74	0.39	0.67	0.32	0.42	0.73	0.66	0.73	0.37	0.38	0.37	0.32	0.75	0.57		0.51
Wolf	0.38	0.71	0.33	0.25	0.36	0.55	0.71	0.35	0.63	0.73	0.35	0.65	0.29	0.39	0.71	0.64	0.71	0.33	0.35	0.33	0.27	0.74	0.59	0.51	

Table S2. Candidate outlier SNPs from BayPass genome scan for life history traits. Candidate loci are shown for each significant trait association. Loci that did not map to a gene are listed a "NA" under "Gene".

Chromosome	Base pair	XtX	Bayes factor (dB)	Trait	Gene	Trait correlation
Omy02	37479696	21.9	21.9	Maximum length	NA	0.69
Omy02	79179404	23.0	23.6	Maximum length	Unclassified	0.42
Omy02	79913459	26.0	37.4	Maximum length	NA	0.74
Omy03	43667880	23.4	28.0	Maximum length	NA	0.51
Omy04	16156017	20.9	20.8	Maximum length	nkain2	0.47
Omy04	16156017	20.9	22.1	Age-1 growth	nkain2	0.44
Omy04	16166581	23.3	32.2	Maximum length	nkain2	0.25
Omy04	16166581	23.3	27.2	Age-1 growth	nkain2	0.67
Omy04	16174986	26.4	34.4	Maximum length	nkain2	0.67
Omy04	16174986	26.4	30.7	Age-1 growth	nkain2	0.27
Omy04	16197311	23.2	23.7	Maximum length	nkain2	0.63
Omy04	16197311	23.2	22.5	Age-1 growth	nkain2	0.26
Omy04	16272317	24.3	25.2	Maximum length	nkain2	0.62
Omy04	16272317	24.3	23.4	Age-1 growth	nkain2	0.27
Omy04	16287433	25.4	28.4	Maximum length	nkain2	0.63
Omy04	16287433	25.4	25.9	Age-1 growth	nkain2	0.20
Omy04	16293879	25.5	22.1	Age-1 growth	NA	0.08
Omy04	16323196	29.9	21.3	Age-1 growth	NA	0.02
Omy06	55776116	29.3	23.8	Age-1 length	NA	0.36
Omy08	14075451	24.0	23.6	Age-1 growth	NA	0.19
Omy09	33993503	21.2	23.5	Age-1 growth	Unclassified	0.35
Omy09	35368324	27.2	28.3	Maximum length	Unclassified	0.17
Omy09	35368324	27.2	21.0	Age-1 growth	Unclassified	0.37
Omy09	35454183	25.0	20.6	Maximum length	Unclassified	0.21
Omy09	35454183	25.0	21.3	Age-1 growth	Unclassified	0.23
Omy11	63819958	24.2	24.0	Maximum length	NA	0.59
Omy12	13253330	21.2	24.2	Maximum length	Unclassified	0.77
Omy12	16592983	20.8	20.1	Age-1 growth	Unclassified	0.01
Omy13	17952825	24.8	24.2	Age-1 growth	NA	0.74
Omy13	17977659	29.5	21.3	Age-1 growth	Unclassified	0.58
Omy15	18422978	22.7	34.0	Maximum length	scn5lab	0.84
Omy16	24692340	27.2	22.6	Maximum length	Coasy	0.30
Omy16	24692340	27.2	21.3	Age-1 growth	Coasy	0.19
Omy16	40594223	28.3	20.8	Maximum length	arhgef25a	-0.31
Omy18	16166892	20.8	22.4	Maximum length	NA	0.69

Omy18	16166892	20.8	27.9	Age-1 growth	NA	0.44
Omy19	36718124	20.5	28.3	Age-1 growth	NA	0.87
Omy19	36726274	22.6	29.8	Age-1 growth	Unclassified	0.87
Omy19	36728226	20.5	27.8	Age-1 growth	Unclassified	0.86
Omy19	36806286	27.2	29.7	Maximum length	Unclassified	0.26
Omy19	36806286	27.2	32.6	Age-1 growth	Unclassified	0.75
Omy27	18675786	20.5	21.6	Age-1 growth	Unclassified	-0.60
Omy28	2485397	22.5	30.0	Maximum length	Unclassified	0.14
Omy28	2485397	22.5	22.0	Age-1 growth	Unclassified	0.67
Omy30	37366790	24.2	22.3	Maximum length	NA	0.46
Omy30	37366790	24.2	21.3	Age-1 growth	NA	0.55
Omy31	15266210	23.8	20.4	Maximum length	NA	0.14
Omy31	27392316	23.8	22.9	Age-1 growth	Unclassified	0.70
OmyY	41386855	28.3	20.6	Age-1 length	NA	-0.72

Table S3. Candidate outlier SNPs from BayPass genome scan for additional XtX outlier loci not associated with a focal life history trait. Loci that did not map to a gene are listed a "NA" under "Gene".

Chromosome	Base pair	XtX	Gene
Omy01	52246123	32.6979389	Unclassified
Omy01	52285804	35.1850256	Unclassified
Omy02	79709053	31.2330875	NA
Omy02	79720006	30.8092152	NA
Omy05	95437059	31.8605703	NA
Omy07	68555374	30.9696087	NA
Omy07	86202350	32.348608	atp2b2
Omy08	75776994	31.0021342	NA
Omy09	35694068	34.3630703	Unclassified
Omy10	180420	31.2145634	Unclassified
Omy10	4230574	38.888181	trpc6a
Omy10	7567427	30.7735481	Unclassified
Omy15	1796655	30.089654	NA
Omy15	1874592	30.3286392	NA
Omy15	13139031	32.8644422	NA
Omy16	20077767	30.2456573	NA
Omy16	20249336	32.1233031	dnah3
Omy18	71895114	31.6210294	Unclassified
Omy27	1002509	32.6734438	col4a4
Omy27	1172388	30.5639448	NA
Omy31	31499070	35.0381045	NA
